# Research Participant Interest in Learning Results of Biomarker Tests for Alzheimer Disease

**DOI:** 10.1001/jamanetworkopen.2025.2919

**Published:** 2025-05-06

**Authors:** Spondita Goswami, Sarah M. Hartz, Amy Oliver, Sacha Jackson, Tomi Ogungbenle, Alissa Evans, Erin Linnenbringer, Krista L. Moulder, John C. Morris, Jessica Mozersky

**Affiliations:** 1 Bioethics Research Center, Washington University in St Louis, St Louis, Missouri; 2 Knight Alzheimer Disease Research Center, Washington University in St Louis, St Louis, Missouri; 3 Department of Psychiatry, Washington University in St Louis, St Louis, Missouri; 4 Department of Health Care Ethics, Saint Louis University, St Louis, Missouri; 5 Division of Public Health Sciences, Washington University in St Louis, St Louis, Missouri

## Abstract

**Importance:**

While available evidence suggests there are not major psychosocial harms of return of research results, there are limited data on uptake of Alzheimer disease (AD) research results or reasons for declining to learn results among participants in AD research studies.

**Objectives:**

To quantitatively and qualitatively evaluate who declines to learn individual AD biomarker research results and what factors are associated with the decision.

**Design, Setting, and Participants:**

This observational cohort study was conducted between November 1, 2020, and April 15, 2024, among participants aged 65 years or older with unimpaired cognition and available biomarker data (apolipoprotein E genotype and either imaging [amyloid positron emission tomography and magnetic resonance imaging] or plasma amyloid level) enrolled in a longitudinal cohort of cognitive aging at the Knight Alzheimer Disease Research Center.

**Exposure:**

Participants with no prior option to receive research results were offered the option to learn these results.

**Main Outcome and Measures:**

The primary outcome was the decision to receive AD research biomarker results. Associations of this decision with demographic factors including self-identified race, parental history of AD, age, gender, and type of biomarker result offered (imaging or plasma) were assessed using χ^2^ tests and semiparametric log-binomial regression. Semistructured qualitative interviews with a subset of participants who declined receiving research results explored reasons for declining.

**Results:**

Of the 274 participants who were offered their research results (mean [SD] age, 75.9 [5.8] years; 158 women [58%]; 35 Black [13%]; and 239 White [87%]), 110 (40%) declined. Black participants were more likely to decline than White participants (adjusted risk ratio, 1.89; 95% CI, 1.43-2.50). Participants with a known parental history of AD dementia were more likely to decline than those without (adjusted risk ratio, 1.49; 95% CI, 1.12-1.98). Qualitative interviews found the following reasons for declining: knowing would be a burden, negative experiences and perceptions of AD dementia, feeling good about memory currently, familial burden, already being prepared, and the uncertainty of results.

**Conclusions and Relevance:**

In this study of participants enrolled in a longitudinal aging study offered their research results, those with a parental history of AD dementia and Black participants were significantly more likely to decline. Qualitative interviews suggest that a family history of AD may create negative experiences and perceptions of the disease, which may influence the decision to learn results. Further research is needed to better understand racial differences in uptake and ensure that the choice to receive research results reflects individual preferences and wishes.

## Key Points

**Question:**

What factors are associated with research participants’ decision to decline learning results of biomarker tests for Alzheimer disease (AD)?

**Findings:**

In this cohort study of 274 individuals with available biomarker data from a longitudinal aging study, 40% of participants declined the option to learn their AD research results; those who self-identified as Black or had a parent with AD dementia were more likely to decline. In a qualitative interview among those who declined, the most common explanation was that knowing would be a burden.

**Meaning:**

These findings suggest that differences in interest in research results should be further explored to ensure adequate communication of the risks and benefits of receiving research results.

## Teaser

This cohort study quantitatively and qualitatively assesses research participants’ decision to learn individual Alzheimer disease biomarker research results and what factors are associated with this decision.

## Introduction

Many Alzheimer disease (AD) research participants want access to their research test results even in the absence of actionability, and there is a growing consensus that return of research results (RoRR) is an appropriate way to respect autonomy.^1,2,3,4,5,6^ However, ethical concerns about the psychosocial harms of learning one is at risk of a highly feared and incurable disease have prevented widespread RoRR for AD research participants.^7,8,9,10,11,12,13,14^ While available evidence suggests there are not major psychosocial harms of RoRR, data are drawn from select populations and long-term follow up are lacking.^7,15,16,17,18,19^

The cautionary approach of no RoRR in AD research is being replaced with the expectation of RoRR. A recent AD research participant bill of rights^3^ strongly advocates for access to AD research results (eg, apolipoprotein E [*APOE*] genotype and amyloid positron emission tomography [PET] results) regardless of cognitive status because these data have personal value, which is in line with recommendations from the National Academies.^4^ Notably, the most recent notice of funding opportunity for the national network of Alzheimer Disease Research Centers (ADRCs) requires that applicants include plans for RoRR, suggesting it is becoming an expectation rather than an exception.^20^ As disease-modifying treatments become available, AD biomarker results generated in research can help determine eligibility for treatments or clinical trials and also predict risk of treatment adverse effects, thereby making previously inactionable results actionable.^21,22,23,24^ Sporadic AD prevention trials—designed for those with evidence of AD neuropathology but without symptoms—necessitate biomarker disclosure.^15,25^

Prior studies consistently indicate a high desire to learn research results regardless of the condition.^2,26,27,28,29,30,31^ One study^2^ of Knight ADRC longitudinal cohort participants found high interest (81%) in RoRR even after participants received education about the limitations of research results. Family history and high subjective risk were both associated with a desire to learn AD research results.^2^

Although stated interest is high, there may be a gap between stated interest and actual uptake. Stated interest in testing for Huntington Disease among eligible individuals was roughly 70% compared with an actual uptake of 17% over a 22-year period.^32,33^ Sanderson et al^34^ found that despite a 63% stated interest in learning lung cancer genetic test results, only 38% of participants chose to learn their results. Some have suggested offering RoRR to individuals could be interpreted as a subtle endorsement to receive them, resulting in hidden harms for participants who declined.^35,36^ However, there is no empirical data on actual uptake of AD research results in a clinical research context or reasons for declining to learn AD research results.

The goal of this study is to evaluate uptake of AD-related research results among participants in a longitudinal study of aging and identify associated factors. To our knowledge, uptake of RoRR has only been studied in familial cancer genetic testing. Two studies^37,38^ found uptake was lower than initially anticipated, and 1 study^38^ found that declining genetic results was associated with younger age, family history of cancer, and being self-identified as a race other than White.

## Methods

### Current Study

This is an observational cohort study of participants in a longitudinal study of aging and dementia who were offered research results as part of a secondary study (Figure). In the informed consent for the longitudinal study, participants were informed they would not receive research results, but demand for results has been high nonetheless.^2,39,40,41^ Given concerns about the psychosocial and cognitive impact of learning research results, we designed a delayed-start randomized clinical trial to evaluate the impact of offering AD biomarker research results. The Washington University Study of Having AD Research Results Explained (WeSHARE) offers participants enrolled in the Knight ADRC longitudinal cohort the option to learn their research results. In this cohort study, we compare participants who declined the offer to learn research results with those who chose to receive the results (ie, consented to the WeSHARE clinical trial). To evaluate factors associated with this choice, we qualitatively interviewed a subset of participants who declined. This observational study of participation in WeSHARE was approved as an expedited study by the Washington University in St Louis institutional review board with a waiver of consent to collect demographic data on those who decline to enroll; the observational study followed Strengthening the Reporting of Observational Studies in Epidemiology (STROBE) reporting guideline. The qualitative study was approved as exempt by the Washington University in Saint Louis institutional review board and participants provided verbal consent before interviews; the qualitative study followed the Standards for Reporting Qualitative Research (SRQR) reporting guideline.^42^

**Figure.**
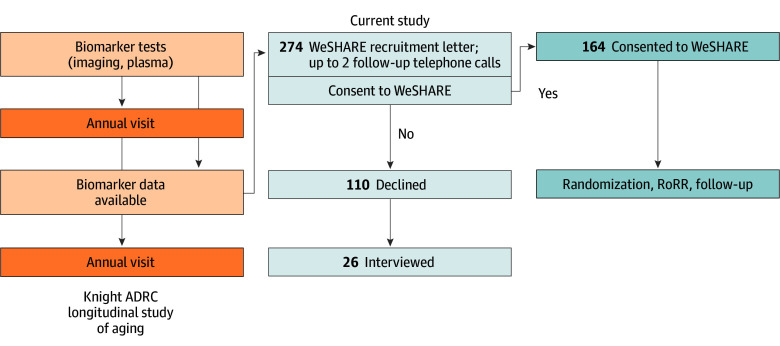
Recruitment of Participants for the Current Study Participants for this study were recruited from the Knight Alzheimer Disease Research Center (ADRC) longitudinal study of aging, where they had consented to participate in future studies but had been told that they would not receive research as part of that study. If participants stated interest in receiving research AD biomarker results, they were enrolled in the Washington University Study of Having Alzheimer Disease Research Results Explained (WeSHARE) clinical trial where they were randomized to delayed-start AD biomarker return of research results (RoRR). Those who declined research results were offered to participate in a qualitative interview.

### Design and Recruitment

Eligible participants were recruited between November 1, 2020, and April 15, 2024, from the Knight ADRC at Washington University in St. Louis. The Knight ADRC longitudinal cohort recruits adults willing and able to undergo longitudinal clinical and cognitive examinations and various biomarker testing including blood, lumbar puncture, magnetic resonance imaging and/or PET. Participants must have a study partner who can serve as their informant and be aged either 65 years or older or younger than 65 years with symptomatic dementia or have a first degree family history of dementia.^43,44,45^ The number of active participants in the cohort varies over time based on attrition and enrollment. At the end of 2024, the active number of participants was 863.

Inclusion criteria for the WeSHARE study were (1) consent for future contact, (2) cognitively unimpaired as measured by a Clinical Dementia Rating ^46,47^ score of 0 at the most recent clinical assessment within 12 months, (3) age 65 years or older, and (4) available AD biomarker research results less than 5 years old (*APOE* genotype and either PET amyloid and magnetic resonance imaging hippocampal volume, or plasma amyloid). The study team had no control over whether a plasma or imaging biomarker result was available.

All eligible participants were contacted by mail informing them of their eligibility for WeSHARE, an educational brochure designed to facilitate decision making, and a WeSHARE consent form to review (Figure).^48^ If a participant had not already contacted us, we telephoned them 2 weeks after mailing the information. Up to 2 telephone messages were left. Participants were categorized as having declined AD research results if they either explicitly declined participation in WeSHARE or they did not respond after the mailing and 2 telephone messages.

Participants who consented to WeSHARE were given their research results and a synthesized 5-year absolute risk of developing the first signs of AD dementia based on their demographics and research results.^48,49,50^ Protocol details are published elsewhere.^50^ Follow-up data collection for those who receive research results is ongoing. Demographic variables were obtained from the Knight ADRC biostatistics core who manage all data collection for the longitudinal cohort.

### Qualitative Study Design and Recruitment

The semistructured in-depth qualitative interviews of participants who declined were conducted 1 to 8 weeks after declining. Interviews examined: (1) decision making and psychological reaction to offer to learn results; (2) reasons for declining; (3) views of memory, health, and future; and (4) attitudes toward research (see the eAppendix in Supplement 1). Interviews lasted 30 to 60 minutes, and participants were paid $40. Two study team members (S.J. and A.E.) conducted the interviews after being trained by one of the principal investigators (J.M.).

### Statistical Analysis

#### Quantitative Data Analysis

We report basic descriptives of those who chose to receive research results and those who declined: self-identified race, AD parental history, age, self-identified gender, and type of biomarker result offered (plasma or imaging). Race was categorized as Black and White. One individual who did not provide race and 1 individual who self-identified as multiracial were included in the group categorized as White. For ethical reasons no participant data were excluded, and this ensures only individuals who self-identified as Black were categorized as Black. Race was included because (1) the Knight ADRC has a longstanding history of increasing enrollment of Black participants who are underepresented in AD research and has been guided by the African American Advisory Board^51^ since 2000 and (2) previous studies have identified racial differences in uptake for other diseases. To examine associations of covariates with the decision to receive results, we used χ^2^ tests for univariate associations and semiparametric log-binomial regression to compute adjusted relative risks (aRRs).^52^ Statistical analyses were performed in SAS 9.4 (SAS Institute).^53^ The threshold for significance was a 2-sided *P* < .05.

#### Qualitative Data Analysis

Qualitative interviews were audio-recorded, transcribed, and deidentified before uploading into Dedoose qualitative analysis software, version 9.2 (SocioCultural Research Consultants).^54^ We used thematic content analysis.^55,56^ Research team members reviewed all transcripts and developed a coding scheme to reflect the 4 interview topics and unanticipated themes emerging. In the first coding phase, a principal investigator (J.M.) read and coded a subset of transcripts to create a preliminary codebook. Three coders (S.G., T.O., and S.J.) used the Dedoose training center to ensure interrater reliability was greater than 0.75 using the Cohen κ coefficient. Additionally, coders coded 2 interview transcripts independently to ensure that the κ coefficients remained greater than 0.75 before coding all remaining transcripts. Coding, including adjustments to the codebook, was an iterative process involving weekly consensus meetings to discuss and resolve coding discrepancies and included an audit trail of coding rules and decisions made. We report the reasons why participants declined to learn their results, their decision-making process, and emotions evoked by the offer. The reasons for declining usually arose spontaneously and were not responses to a direct question.

## Results

### Quantitative Results

Characteristics of participants who enrolled in, and declined, the WeSHARE study to learn their results are reported in Table 1. Of the 274 participants who were offered results (mean [SD] age, 75.9 [5.8] years; 158 women [58%]; 35 Black [13%]; 239 White [87%]), 110 [40%] declined results and 164 [60%] consented to results. There were significantly more Black participants who declined results (24 participants [68%]) than White participants (86 participants [36%]) (χ^2^_1_ = 14.03; *P* < .001). Other characteristics of those more likely to decline their research results were parental history of AD dementia (67 of 144 participants with a parental history [47%] vs 43 of 130 participants without [33%]; χ^2^_1_ = 5.53; *P* = .02) and the offer of plasma biomarker results (46 of 86 participants offered plasma [53%] vs 64 of 188 participants offered imaging results [34%]; χ^2^_1_ = 10.04; *P* = .002). After adjusting for the reported covariates, Black participants continued to be significantly more likely to decline research results than White participants (aRR, 1.89; 95% CI, 1.43-2.50), and participants with a known parental history of AD dementia were more likely to decline research results than participants without (aRR, 1.49; 95% CI, 1.12-1.98). After adjustment, participants offered plasma biomarkers were no longer statistically less likely to decline RoRR than participants offered imaging biomarkers. Age was not statistically significant, although there were more adults in older age groups than younger age groups who declined results (12 of 41 participants aged 65-69 years [29%] vs 36 of 73 participants aged 80 years or older [49%]).

**Table 1.** Demographic Characteristics of Samples and Adjusted Relative Risks of Declining Research Results

Characteristic	Quantitative sample			Declining results, aRR (95% CI)^a^	*P* value	Declined results and completed qualitative interview, No. (%) (n = 26)
	Total cohort, No. (%) (N = 274)	Consented to results, No./total No. (%) (n = 164)	Declined results, No./total No. (%) (n = 110)			
Age group, y						
65-69	41 (15)	29/41 (71)	12/41 (29)	1 [Reference]	NA	4 (15)
70-74	80 (29)	54/80 (67)	26/80 (33)	1.03 (0.59-1.80)	.92	4 (15)
75-79	80 (29)	44/80 (55)	36/80 (45)	1.45 (0.85-2.46)	.17	10 (38)
≥80	73 (27)	37/73 (51)	36/73 (49)	1.48 (0.85-2.58)	.17	8 (31)
Gender						
Woman	158 (58)	90/158 (57)	68/158 (43)	1 [Reference]	NA	16 (62)
Man	116 (42)	74/116 (64)	42/116 (36)	0.85 (0.64-1.14)	.28	10 (38)
Race^b^						
Black	35 (13)	11/35 (32)	24/35 (68)	1.89 (1.43-2.50)	<.001	3 (12)
White	239 (87)	153/239 (64)	86/239 (36)	1 [Reference]	NA	23 (88)
AD in parent						
No known parental history of AD	130 (47)	87/130 (67)	43/130 (33)	1 [Reference]	NA	11 (42)
At least 1 parent with AD	144 (53)	77/144 (53)	67/144 (47)	1.49 (1.12-1.98)	.006	15 (58)
Biomarker						
Imaging	188 (69)	124/188 (66)	64/188 (34)	1 [Reference]	NA	19 (73)
Plasma	86 (31)	40/86 (47)	46/86 (53)	1.28 (0.94-1.75)	.12	7 (27)

Abbreviations: AD, Alzheimer disease; aRR, adjusted risk ratio; NA, not applicable.

^a^ aRRs are adjusted for all variables in the table.

^b^ One person who identified as multiracial and 1 who did not provide their race were included among the 239 White individuals.

### Qualitative Results

Of the 110 participants who declined to participate in WeSHARE, we reached 60 by telephone and asked whether they would participate in a qualitative interview; the remaining 49 participants who declined to participate in WeSHARE did not answer our telephone calls. Thirty participants stated they would participate in a qualitative interview. There were 2 Black participants who were lost to follow-up due to a staffing change and 2 White participants were not asked because we had reached thematic saturation. In total, we interviewed 26 study participants (10 aged 75-79 years [38%]; 16 women [62%]; 3 Black [12%]; 23 White [88%]) about declining to learn their results. We asked participants about their decision-making process including if they spoke to others and whether the offer evoked emotions; most (17 participants) spoke to others. When asked if the offer evoked emotions, a subset (9 participants) indicated it had caused some mild worry, anxiety, or thoughts about AD, but no significant negative emotions (ie, serious, long-lasting emotions that caused distress or harm due to the offer of learning results) were reported after being offered results. We asked participants if they might change their mind in future. The majority (20 participants) indicated they might change their mind; primary reasons for this were if they developed memory concerns (6 participants), the availability of prevention treatment (7 participants), or a change in attitude about receiving results (5 participants).

The most common theme for declining results (21 participants) was that learning results would be a burden and could exacerbate or create new worries about their own memory and the possibility of getting AD dementia. This included more generalized concerns about the burdens of knowing such as changing how they lived their lives, altering relationships, or disrupting current well-being (Table 2).

**Table 2.** Perceived Burdens of Knowing Results

Perceived burden	Illustrative quotation
Create or exacerbate memory concerns	“Then when I was actually faced with the possibility of getting them, I started to do more of a deep dive into that, and I realized that I think I would live my life with some hesitation or doubt or something like that if I was always wondering if I forgot the name of someone or the direction somewhere, that I’d be like, ‘Oh my gosh, this is it. It started. I’m on the trajectory.’ For all I know, I’m already on the trajectory. I don’t know, but I think I would be more concerned and I’d have more self-doubt and I would worry more if I had the results.” (age group, 65-69 years; White; woman; P17)
	“…I felt like I would be thinking that every little thing that happened, that was what was happening was that I was developing Alzheimer’s. I just at the present time just decided not to do it.” (age group, 85-89 years; White; woman; P18)
Knowing creates negative emotions	“For me, I think it would be very negative and disruptive to my life to know that I was going to get…or the chances were good, based upon this research. To know that I was going to get Alzheimer’s would be very disturbing to me. I think it would…I don’t need to know. That’s like saying, ‘Well, you know, if you walked across a street and a car hits you, you’re gonna die.’ Why would I wanna know that? Why would I wanna have that in my head for the rest of my life? For me, it would not be a good idea at all.” (age group, 70-74 years; White; woman; P21).
Might change how I live my life	“I thought, well, if I knew when I would get dementia or the possibility that I would get dementia, would that change my life? Would I start doing things differently any better what I’m doing now? My answer was a resounding yes. I think my whole life would probably change. My relationships with others would probably change. I thought maybe I just will not know.” (age group, 75-79 years; Black; man; P14)
	“If the research results suggested that I was more likely to get it, I guess I just thought that…I think it would probably affect the way I do things. I guess I just didn’t want to take a chance on having my life changed or altered because of something I knew about my health.” (age group, 80-84 years; White; man, P2)

Abbreviation: P, participant.

The second most common theme (20 participants) involved unprompted and negative descriptions of prior experiences and perceptions of AD, with participants using words such as “horrible,” “very difficult,” or “very hard to watch.” This encompassed experiences caring for or knowing others with AD dementia, and evaluative descriptions of how they perceived AD dementia (Table 3). Two primary subthemes emerged: the pain of witnessing cognitive decline and the burdens of caregiving. Two participants expressed more extreme views that death or assisted suicide would be preferable to developing AD dementia based on their experiences.

**Table 3.** Experiences and Perception of Alzheimer Disease Dementia

Experience	Illustrative quotation
Negative experiences and perceptions	“I think I let you know how terrible a disease I think it is, that it has finally affected my life through my friends and in-laws. That’s hit. It’s made it hit home even more in the last couple of years. I guess I need to say that.” (age group 70-74 years; White; woman; P5)
	“…Oh, it was so sad...It is a horrible disease…it’s a terrible diagnosis.” (age group, 80-84 years; White; woman; P25)
Pain of witnessing cognitive changes or decline	“We have friends…my best friend’s husband has…He has no short-term memory whatsoever…and he’s getting worse. …I watch him and I see, and I see the grief it causes my friend. My sister-in-law also in the last two years was diagnosed with dementia and she’s now in a memory care facility, and I see what it’s done to the family. …It’s that middle part in between when you’re still somewhat cognizant and unknowing that’s a terrible part of life, and that’s what my friend and my sister-in-law are going through.” (age group, 70-74 years; White; woman; P5)
	“I think the other thing is, I have a good friend that I was just told that he has stage four dementia. Our conversations have really changed a lot. Because now the only thing that he wants to talk about is stuff that happened way back before I even knew him. That’s our basic conversations. Plus, his daughters have moved him from [City 1], [State 1], to [City 2], [State 1], and he keeps telling me about how he hates that city. That’s all that he talks about. That, as well as, mainly, like I said, stuff that happened from years and years and years ago.” (age group, 75-79 years; Black; man; P14)
Burdens of caregiving	“My father had Alzheimer’s for more than ten years. My brother and I took care of him. It was not pleasant. He had one sibling...She also got Alzheimer’s…but that’s a concern to both me and my brother. I really wouldn’t want to know.” (age group, 70-74 years, White, woman, P21)
	“ …I would be worried about how my husband would be able to take care of me, my children, because I saw how it affected my own mother-in-law when my father-in-law developed Alzheimer’s. It was very, very hard to watch. I just wouldn’t wish that on anybody to have to go through all that. I learned a great deal from watching my mother-in-law. She’s a very strong woman. Oh, she was just very stoic through it all taking care of her husband.” (age group 70-74 years; White; woman; P11)

Abbreviation: P, participant.

The remaining themes are described in Table 4. These include lack of treatments and limited utility (16 participants), feeling good about memory currently (12 participants), burden to family and loved ones (9 participants), already having prepared for the future (8 participants), and the uncertainty of the information (8 participants). Two remaining themes arose infrequently: concerns about insurance discrimination (2 participants) and being treated differently by others (2 participants).

**Table 4.** Additional Reasons for Declining Research Results

Reason for declining results	Illustrative quotation
Lack of treatments or utility	“I can’t change it. If they said, If you take this test and you’ve got it, we can do something to change it, then I would be more interested.” (age group, 75-79 years; White; woman; P8)
	“ …if you did find out you had dementia, there’s really…I know there’s a couple of new drugs out there that are supposed to slow it down, but there’s really no cure. I felt like, well, now you know you have it, or you might get it. What are you gonna do about it?” (age group, 75-79 years; White; man; P13)
	“On the other side of it, if I did find out that there was something wrong, I don’t know that I would do anything particularly different at the moment. I’m adverse at taking medicines. … I think I’m in good shape right now…” (age group, 75-79 years; White; man; P4)
I feel good about my memory	“No, not really. Maybe if I had been concerned about my memory, if I had seen some reason to be concerned, perhaps, but I don’t see any reason to be concerned about my memory in terms of day-to-day functions or ability to make decisions and handle problems, and all of that. No.” (age group, 65-69 years; Black; man; P26)
	“I felt pretty confident that I wouldn’t be getting any dementia in the next five years. I guess, like I said, I was concerned that if I got some information that was negative…then you’re thinkin, well, when’s the hammer gonna come down on you now that you got this potential information that says you may be a candidate to get it when right now I feel like all signs are that I’m not gonna be a candidate at least in the next five years.” (age group, 75-79 years; White; man; P13)
Knowing would burden my family and loved ones	“No because I honestly for years thought I wanted to. I wanted to and then it hit me in the face that here it was in my face, that here was the opportunity and I just couldn’t do it. I guess maybe I will say I’m too afraid. It’s okay. I don’t know, but I don’t want to put my family through any because they will all…if we said today that I’m going to get my results and if I came home and said, ‘Well, it looks like maybe I might be having some dementia coming on in the next five years,’ they would all be so upset and worried and I don’t want to do that to them.” (age group, 70-74 years; White; woman; P5)
	“That’s what it boiled down to, putting…especially me subjecting her to something that may or may not be. Why do that? Just so much uncertainty and just unnecessary burden on someone.” (age group, 65-69 years; Black; man; P1)
I have already prepared all that I can	“Basically, if indeed I do wind up in memory care, that’s covered, number one, by our residence in the continuing care community, or continuing care retirement community. Number two, it’s backed up by my long-term care insurance. I think we’re in pretty good shape. We did our wills about 10 years ago. Not wills. I’m sorry. We did our funerals about 10 years ago. Wills were done a long time ago. I guess we’re planners.” (age group, 75-79 years; White; man; P22)
Uncertainty of information	“It could be, ‘Hey, your chances are slim to none.’ Or it could be, ‘Well, we’ve seen this or seen that, but still that doesn’t mean that you’re gonna get that.’ Why enter all those uncertain factors into it? That was our baseline reason for saying, we appreciate it, but no.” (age group, 65-69 years; Black; man; P26)

Abbreviation: P, participant.

## Discussion

To our knowledge, this cohort study is the first to evaluate who declines to learn individual AD research results and examine the reasons for doing so. We had lower than expected interest, with 60% choosing to learn their results, compared with 81% prior stated interest in the same cohort.^2^ Although unexpected, this higher rate of declining provides some reassurance that our approach and educational materials, which included pros and cons and a decision-making worksheet, provided balanced information that enabled participants to decline; this underscores the ethical importance of respecting the right not to know and ensuring individuals have a choice to decline learning their research results. It also highlights the difference between stated intentions and actual behaviors when faced with the choice to learn one’s AD research results.

Our study involves individuals already enrolled in a longitudinal research study but who choose specifically not to learn their AD biomarker results when offered. Thus, reasons for declining to learn AD biomarker results among this group are less likely to reflect attitudes toward research, but rather specifically relate to not wanting to learn information about their personal risk of developing AD. This study expands upon existing literature, which has focused primarily on declining to enroll in research altogether.^57^

We saw that there were more participants in older age groups who declined to learn their results, although this finding was not statistically significant; this may be due to 5-year risk of developing the first signs of AD being less relevant as people age, or conversely that learning one’s risk may be of greater concern as people get closer to the potential of age of onset. While not statistically significant after adjusting for covariates, there were more participants who declined results when offered plasma results. Our team had no control over which biomarker result was available to offer, but further research is needed to ensure there are not differences in attitudes about the value of blood-based biomarkers as opposed to imaging.

We identified significant racial differences in the decision to receive research results, with Black participants being significantly more likely to decline AD biomarker research results compared with White participants. The underlying reasons for this racial disparity are not known, and our qualitative data do not suggest differences in reasons for declining among Black and White interviewees. However, Black participants may have more concerns about learning their results given they disproportionately experience stress, discrimination, and stigma relative to White participants.^58,59^ Rahman et al^6^ found high hypothetical interest in learning research results among both Black and White participants, but Black care partners were less interested. Further research is needed to better understand differences by race and to ensure that our approaches, processes, and materials are culturally appropriate so that we identify and overcome any systematic issues that may lead to differences in uptake by race.

Additionally, parental history of AD dementia was significantly associated with declining to learn results. Contrary to existing data that family history is associated with an increased desire to know, individuals with a parental history of AD were more likely to decline RoRR.^2,60^ While family history may be an important factor in the decision to join AD research, our data suggest this does not necessarily translate to wanting to learn one’s risk of developing the disease. Promoting RoRR as an incentive to join research could potentially be a deterrent for some and highlights the importance of individual choice. Our qualitative findings help elucidate this discrepancy because many individuals described negative experiences and perceptions of AD that likely contributed to their view that learning their risk of developing AD would be burdensome and anxiety-inducing rather than empowering. The most common reason for declining was that it would create or exacerbate concerns about memory and developing AD dementia, which is likely especially worrisome given participants’ prior experiences.

The primary reasons for declining identified in qualitative interviews were consistent with prior literature and suggest that the potential to create or exacerbate worry affecting how people live their lives, burdening loved ones, and a lack of actionability remain important reasons for not wanting to know. This finding suggests that the availability of disease-modifying treatments for asymptomatic individuals will increase individual willingness to know, but educational materials that convey the option to not learn research results will still be needed going forward to enable choices that respect individual autonomy. Materials that address the most common concerns such as exacerbating memory concerns or burdening loved ones may help individuals make informed choices. Another common reason individuals did not want to know their results was that they were not experiencing any memory issues currently, which is notable given that prior hypothetical studies have found an association between subjective memory concerns and wanting to know.^2^ Individuals in our study did not want to disturb their current well-being which was attributable in part to not having memory concerns currently, and a common reason they might change their mind was if they developed memory concerns in future.

### Limitations

This study has limitations. Participants were already part of a longitudinal cohort, indicating a pre-existing level of trust and willingness to participate in research that might be different from the general population. This pre-existing trust is also a strength because it allows us to specifically examine the reasons behind participants’ decision not to learn their results. Participants had higher education and socioeconomic status compared with the general population, which limits generalizability and may explain the lack of concerns about insurance and other forms of potential discrimination seen in qualitative interviews.^61^ Participants were also likely to be more interested in cognition which also limits generalizability. Although we did not measure health literacy, our population is highly familiar with and knowledgeable about AD, raising questions about how those with low health literacy and little knowledge about AD may respond to the offer of AD research results. Our qualitative sample consisted predominantly of White individuals, with only 3 Black participants. More research is needed to better understand reasons for declining to learn research results among self-identified Black participants because declining to take part in a qualitative interview may reflect lower overall engagement with research.

## Conclusions

Our study findings underscore the importance of respecting the right not to know given that 40% of participants enrolled in a longitudinal cohort chose not to learn their AD research results when offered. Race and family history were significantly associated with declining to receive results. Qualitative findings reveal how negative experiences and perceptions of AD dementia affect individual’s desire to know and help elucidate why family history is negatively associated with wanting to know. Future research should investigate racial differences in decision to learn results; this is necessary to identify addressable factors that may impact racial differences in uptake and ensure we are respecting individual autonomy.

## Notes

Supplement 1. **eAppendix.** WeSHARE Decline RoRR Group—Qualitative Interview Guide

Supplement 2.
Data Sharing Statement
