# Supplementary material for: Research Participant Interest in Learning Results of Biomarker Tests for Alzheimer Disease
Source: JAMA Netw Open. 2025 May 6;8(5):e252919. doi: 10.1001/jamanetworkopen.2025.2919 (PMC12056564; doi:10.1001/jamanetworkopen.2025.2919)
Supplement: Supplement 1. — eAppendix. WeSHARE Decline RoRR Group—Qualitative Interview Guide [file jamanetwopen-e252919-s001.pdf]

Supplemental Online Content

Goswami S, Hartz SM, Oliver A, et al. Participant interest in learning results of research biomarker tests for Alzheimer disease. *JAMA Netw. Open*. 2025;8(3):e252919. doi:10.1001/jamanetworkopen.2025.2919

eAppendix. WeSHARE Decline RoRR Group—Qualitative Interview Guide

This supplemental material has been provided by the authors to give readers additional information about their work.

© 2025 Goswami S et al. *JAMA Network Open*.

eAppendix. WeSHARE Decline RoRR Group—Qualitative Interview Guide

Introduction:

Read Exempt Information Sheet Decliner RoRR_2024 06-09.

Do you have any questions for me? [answer any questions]. Is it OK if I start recording now?

**Stem**	**Probes [***Not all probes will be needed as a participant may spontaneously answer something before it is asked or it may not be necessary to follow up. Probes labeled with* **[O]** *are optional and primarily to help if someone has difficulties answering a question by restating the question differently]*	**Constructs addressed**
**Background opinions and interest in Research Results**<br>*I'd like to start by asking you about your interest and decision about getting your research results.*		
1. Can you tell me a bit about how you made the decision to not get your results? [i.e. easy, had to really deliberate etc.]	• Did you ask anyone for advice or input about your decision?<br>• Did making the decision bring up any emotions for you, positive or negative?<br>• Did you have a chance to read the educational brochure before making your decision?<br>○ ***[If yes]:*** How did you find the information in the educational brochure?<br>○ Did it help you decide? And if yes, how?	Autonomy and right to know or not know
2. Why did you decide not to receive your research results?	• Did you have any concerns about what might happen if you learned your results?<br>*[Depending on how much participant tells you initially, probe the following]*:<br>• Did you have concerns about how knowing your results could affect you or how you views your future?<br>• Did you have concerns about how others would treat you?<br>• Did you have concerns about insurance?<br>• Did you have concerns about lack of treatments for AD dementia?<br>• Anything else?<br>• In general, what do you think about genetic testing?<br>○ And what do you think about other tests, like brain imaging results?<br>• Can you tell me a bit about what you have heard about new treatments [or other tests or advancements] for AD?<br>○ Do these treatments have any impact on how you think or feel about learning your results?	Personal utility, reasons for declining, psychosocial harms and benefits

© 2025 Goswami S et al. *JAMA Network Open.*

	o Anything else you would like to tell me about new treatments for AD? [Continue to probe as needed depending on participant response]<br>• [Statements about new treatments, testing, or advancements in AD dementia may arise spontaneously at any point in the interview – address these questions when they arise. If topics do not arise sponteaneously, ask them here.]	
**Participant reactions to their decision about receiving research results.**<br>*Thank you. So now I would like to ask some questions about how you felt after you made your decision.*		
3. How much has your decision been on your mind since you decided not to get your results?	• **[O]** Can you tell me more about that?<br>• Do you think you might change your mind about receiving results?<br>o [If yes] What might lead you to change your mind?	
**Participant attitudes about memory, health, and the future**		
4. How do you feel about your health and memory compared to most people your age?	• Can you tell me more about that?<br>• Do you do anything currently, such as exercise, diet, brain games, to try to reduce your risk of getting AD dementia?<br>• Do you have plans in places for the future like longterm care insurance, or any other plans in case you get dementia in the future?	Views of health, memory and future
5. How do you feel about your future?	• **[O]** Can you tell me more about that?	
**Views regarding research.**<br>*We are nearing the end of my questions. Next, I want to ask you a little bit about participating in this study and other MAP studies.*		
6. How do you feel overall about your participation in MAP?	• Is there anything that would make you more interested in staying in MAP?	Impact on research participation
**Anything Missed?**		
7. That was my last question. Is there anything we didn't talk about that you want to mention? Do you have any questions for me?		

Closing: Thanks again for taking the time to talk with me. Your willingness to talk will be very helpful for our study team. [If participant mentioned any topics that merit correction/clarification, say: "I want to mention something that came to mind while we were talking." And then clarify the misconception.] Please call us at XXX-XXX-XXXX if you have any questions about this study or about your research results. As a reminder, we will send you a $40 payment to thank you for your time today. I will need some details from you to arrange the payment, so I am going to turn off the recorder and ask you for some information.

© 2025 Goswami S et al. *JAMA Network Open.*
