# Supplementary material for: Research Participant Interest in Learning Results of Biomarker Tests for Alzheimer Disease
Source: JAMA Netw Open. 2025 May 6;8(5):e252919. doi: 10.1001/jamanetworkopen.2025.2919 (PMC12056564; doi:10.1001/jamanetworkopen.2025.2919)
Supplement: Supplement 2. — Data Sharing Statement [file jamanetwopen-e252919-s002.pdf]

Data Sharing Statement

Goswami. Participant Interest in Learning Results of Research Biomarker Tests for Alzheimer Disease. *JAMA Netw Open*. Published March 28, 2025.
doi:10.1001/jamanetworkopen.2025.2919

Data
Data available: Yes
Data types: Deidentified participant data
How to access data: Data collection is still ongoing for the parent trial and will be made available at the end of the study period via the Knight ADRC. Knight ADRC data can be requested through the online request portal on the Knight ADRC website: https://knightadrc.wustl.edu/professionals-clinicians/request-center-resources/. Qualitative data collection is ongoing. Qualitative data will be deposited at the qualitative data repository at the university of Syracuse – https://qdr.syr.edu – under restricted access due to sensitivity of qualitative data at the end of the study.
When available: beginning date: 05-31-2027

Supporting Documents
Document types: None

Additional Information
Who can access the data: Quantitative data will be made available to other researchers after review and approval by the Knight ADRC. Qualitative data will be available to those who apply and are approved by the QDR repository.
Types of analyses: Analyses must be for specified purposes and approved by the relevant oversight body.
Mechanisms of data availability: Signed data access agreement.
Any additional restrictions: Qualitative data will be available under restricted access only due to sensitivity.
